# Capecitabine-Induced Terminal Ileitis: Case Report and Literature Review

**DOI:** 10.7759/cureus.14621

**Published:** 2021-04-21

**Authors:** Artsiom Klimko, Cristian G Tieranu, Andrei O Olteanu, Carmen M Preda, Elena M Ionescu

**Affiliations:** 1 Division of Physiology and Neuroscience, University of Medicine and Pharmacy "Carol Davila", Bucharest, ROU; 2 Gastroenterology, “Elias” Emergency University Hospital, Bucharest, ROU; 3 Gastroenterology, University of Medicine and Pharmacy "Carol Davila", Bucharest, ROU; 4 Gastroenterology, Fundeni Clinical Institute, Bucharest, ROU

**Keywords:** capecitabine, ileitis, diarrhea, gastrointestinal injury

## Abstract

Capecitabine is a well-established agent for adjuvant chemotherapy in breast and colorectal cancers. However, one of the limiting adverse events of this therapy is severe diarrhea, which is reported with increasing frequency as of late. Capecitabine-induced ileitis should be suspected in cases with severe, treatment-refractory diarrhea. We present a case of capecitabine-induced terminal ileitis in a patient who received the medication as adjuvant therapy for previously resected colon adenocarcinoma. Capecitabine-induced diarrhea secondary to ileitis is a severe adverse drug event, which occurs during adjuvant chemotherapy and does not respond to conservative treatment with antidiarrheals, often necessitating permanent drug withdrawal. A high index of suspicion is critical as the complications, such as dehydration and the associated electrolyte derangements, may be life-threatening if diagnosis and cause-specific treatment are delayed.

## Introduction

Fluoropyrimidines (e.g., capecitabine, 5-fluorouracil (5-FU), trifluridine-tipiracil) are chemotherapy agents, which are cytotoxic and/or cytostatic antimetabolites - they antagonize pyrimidine analog production to halt deoxyribonucleic acid synthesis and induce cell death. The oral agent capecitabine emerged as an attractive alternative to the parenteral 5-FU due to ease of administration, a more favorable side effect profile, and matched efficacy [1]. Capecitabine is frequently used as an adjunctive or palliative agent for locally advanced or metastatic colon and breast cancer. The most commonly reported side effects are myelosuppression, palmar-plantar erythrodysesthesia, and diarrhea.

Chemotherapy-related diarrhea (CRD) is a serious dose-limiting adverse drug reaction, which can be life-threatening and require hospitalization for supportive care. This is particularly relevant for capecitabine, as randomized trial data reported the frequency of severe diarrhea (grade 3 or 4) to be as high as 47% in certain regimens [2]. In rare cases, fluoropyrimidines have been associated with various gastrointestinal tract injuries, such as mucositis, enterocolitis, and, rarely, ileitis [2]. We report a rare case of capecitabine-induced terminal ileitis. We further conduct a literature review to identify patient characteristics and treatment considerations, as the management approach for severe diarrhea secondary to terminal ileitis poses a significant clinical challenge.

## Case presentation

A 68-year-old male presented to the emergency room (ER) of “Elias” Emergency University Hospital in Bucharest with severe watery diarrhea (grade 4), associated with nausea, vomiting, and malaise. Ten days prior, treatment with adjuvant capecitabine was initiated for colon adenocarcinoma. His medical history was significant for diabetes mellitus type 2 treated with long-acting insulin and oral antidiabetic agents and arterial hypertension treated with calcium channel blockers and beta-blockers. The patient denied smoking or illicit drug use. He suffered from a bilateral inguinal hernia, and he was previously diagnosed with moderately differentiated ascending colon adenocarcinoma, which was treated with a right hemicolectomy and ileal-transverse anastomosis.

**Figure 1:**
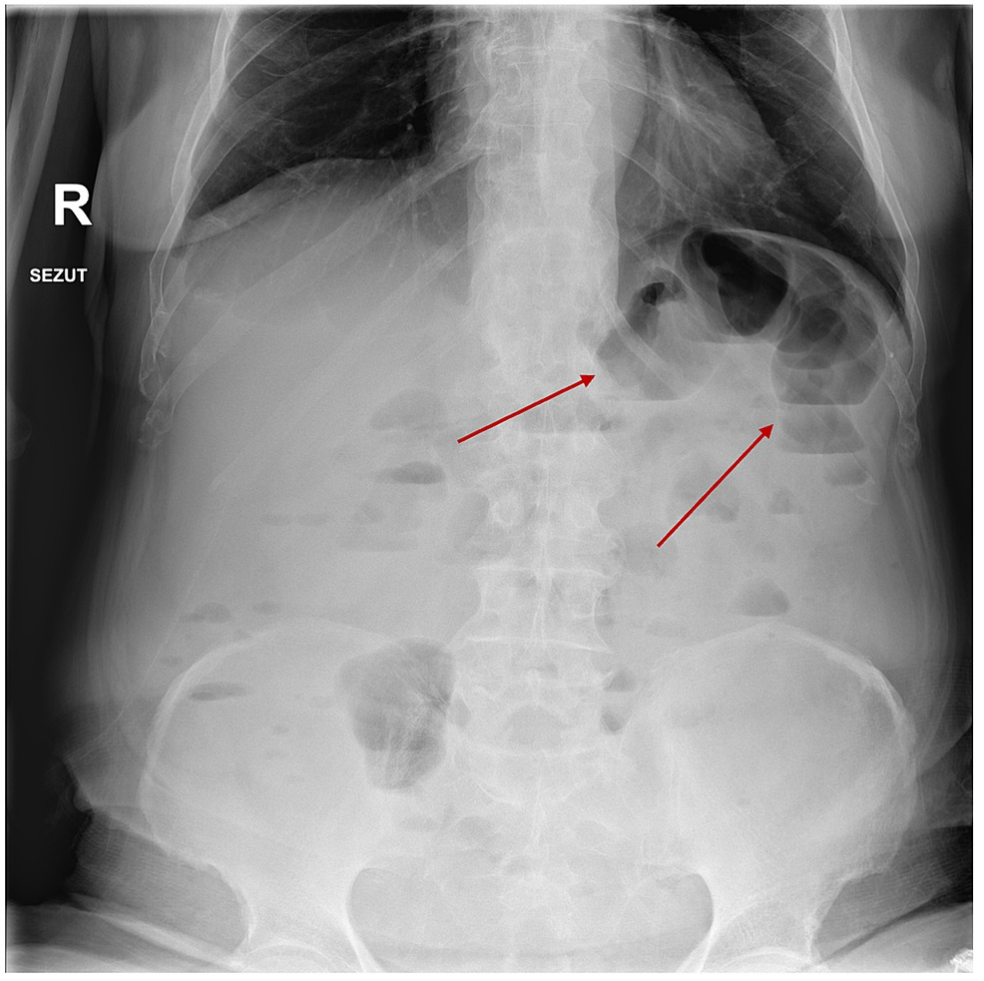
Abdominal radiography conducted at admission showing air-fluid levels (red arrows).

His clinical examination was significant for mild but diffuse abdominal pain exacerbated by palpation, without peritoneal signs. Laboratory examinations were significant for mild hypernatremia (146 mmol/L, reference 135-145 mmol/L), hypokalemia (2.5 mmol/L, reference 3.5-5 mmol/L), serum osmolarity of 299 mOsm/kg, hypoalbuminemia (2.8 mg/dL, reference 3.3-5 mg/dL), slightly elevated blood urea nitrogen, mild anemia (10.5 g/dL, reference 12-15 g/dL) on complete blood count, elevated inflammatory markers (C reactive protein of 46 mg/dL, normal value <5 mg/dL), normal procalcitonin, and normal liver function tests. Exhaustive stool workup did not identify any pathogens, with significantly elevated fecal calprotectin (1053 mcg/g, normal value <50 mcg/g). The abdominal ultrasound at the ER was inconclusive, while abdominal plain radiographs showed several non-specific, small bowel air-fluid levels (Figure 1).

**Figure 2:**
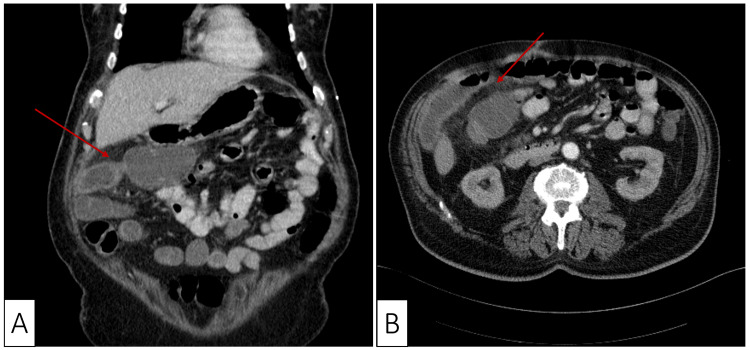
Coronal (A) and axial (B) computed tomography scan demonstrating thickening of the terminal ileum and mural thickening (red arrows).

The patient was transferred to the oncology department where a computed tomography (CT) scan of the abdomen was performed to further evaluate the cause of the air-fluid levels. The result showed ileal wall thickening with contrast enhancement of the adjacent fat and intraluminal vessel enhancement suggestive of a local inflammatory process (Figure 2).

**Figure 3:**
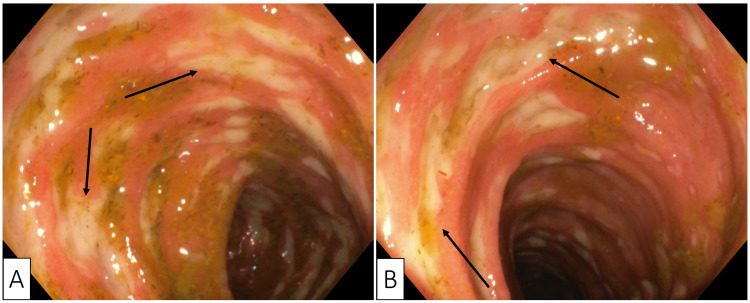
Colonoscopy showing the ileum at the ileo-colonic anastomosis (A) and the terminal ileum (B) with diffusely erythematous mucosa covered with multiple large ulcerations (black arrows).

Colonoscopy revealed a normal ileocolonic anastomosis; however, multiple large ulcers and diffuse erythematous ileitis were discovered, extending approximately 10 cm proximal to the anastomosis (Figure 3). Antidiarrheal drugs were prescribed, consisting initially with loperamide and later with octreotide. However, after three days of treatment there was no significant reduction in stool frequency or improvement of stool consistency. Therefore, capecitabine was withdrawn while continuing loperamide. The patient began to improve clinically. Three days later, gastrointestinal transit was normalized, and the patient was discharged with recommendations to taper down the dose of loperamide.

The patient was tested in the outpatient care clinic for dihydropyrimidine dehydrogenase (DPD) deficiency being heterozygous for the splice-site mutation affecting dihydropyrimidine dehydrogenase gene (DPYD)*2A. Due to the associated costs, the patient declined preemptive DPD screening. Given the relapse risk associated with retreatment in the setting of DPD deficiency, and the adjuvant role of chemotherapy in this case, we decided to discontinue capecitabine permanently and closely monitor the patient further.

## Discussion

Diarrhea is a well-established side effect of capecitabine treatment, usually starting after two to three weeks from the first drug administration, is mostly mild in severity, and rarely necessitates antimotility agents, such as loperamide, for symptomatic control. However, grade 3-4 diarrhea can occur in 11.4% of patients and can be a life-threatening complication in the setting of DPD deficiency [3,4]. Capecitabine causes ileitis presumably through inducing loss of surface epithelium secondary to acute mucosal injury through a mechanism that involves interference with crypt cell mitoses. This leads to a high-volume fluid loss in the small bowel, which exceeds the colonic capacity for reabsorption [2,5].

In order to further identify patient characteristics, management considerations, and outcome trajectories, we conduct a search of the PubMed and Scopus databases for cases describing terminal ileitis induced by capecitabine. A set of keywords was identified either within the title/abstract or as Medical Subject Heading terms. In addition to the eight published articles that were reviewed, four cases of capecitabine-induced terminal ileitis were reported as posters in congresses. Although they were excluded from our review due to being unpublished, they suggest that the prevalence of capecitabine-induced ileitis may be underreported. Colonoscopy and histopathology findings were included in six and four studies, respectively. A total of eight studies was included in our review, totaling 13 patients - our results are presented in Table 1. 

**Table 1:** Published literature describing capecitabine-induced ileitis CT: computed tomography; IVF: intravenous fluids; MRE: magnetic resonance enterography; CAPOX: capecitabine with oxaliplatin; CAPIRI: capecitabine with irinotecan; TPN: total parenteral nutrition

Author and year	Patient gender and age (years)	Chemotherapy regimen prior to diagnosis	Symptoms	Diagnostic findings	Treatment and outcome
Our patient	Male, 68	Adjuvant capecitabine	Severe watery diarrhea (grade 4) accompanied by nausea, vomiting, and malaise	CT - ileal wall thickening with adjacent fat stranding; endoscopy - multiple large ulcers of the ileum with and diffusely erythematous mucosa	IVF, antidiarrheal drugs; diarrhea did not respond to antidiarrheal therapy, but improved within three days of capecitabine discontinuation
Dao et al., 2019 [6]	Female, 72	Neoadjuvant capecitabine	Severe watery diarrhea	CT - mildly dilated fluid-filled bowel loops with mesenteric edema; endoscopy - ulceration of ileum/ileocecal valve and granular erythematous mucosa of ascending colon	IVF, broad-spectrum antibiotics, loperamide, diphenoxylate-atropine, octreotide, cholestyramine; diarrhea resolved after four weeks
	Female, 42	Neoadjuvant capecitabine	Severe voluminous bloody diarrhea (grade 3)	CT - marked wall thickening distal small bowel loops with luminal fluid; endoscopy - severe ileal ulceration with necrosis, bleeding, and pseudomembrane formation	IVF, broad-spectrum antibiotics, loperamide, diphenoxylate/atropine, octreotide, cholestyramine, dietary changes; diarrhea resolved after four weeks
Van Hellemond et al., 2018 [7]	Female, 69	CAPOX	Severe watery diarrhea (grade 4)	MRE - 5 mm thickening of the distal 15 cm of the ileum; endoscopy - superficial, yet extensive ulceration of the terminal ileum; histopathology - extensive inflammation consistent with ileitis	IVF, budesonide; diarrhea resolved
Nicosia et al., 2018 [8]	Female, 71	Combined radiotherapy and capecitabine	Severe watery diarrhea (grade 3)	CT - diffuse edema of the distal ileum with luminal narrowing	IVF, broad-spectrum antibiotics; diarrhea resolved after 15 days
	Female, 54	Combined radiotherapy and capecitabine	Severe watery diarrhea (grade 2)	CT - ileal distension with edematous thickening and perivisceral effusion	IVF, broad-spectrum antibiotics; diarrhea resolved after seven days
Lee et al., 2015 [9]	Female, 61	CAPIRI and bevacizumab	Watery diarrhea, right lower quadrant abdominal pain, vomiting, fever	CT - extensive submucosal ileal edema with adjacent fat stranding	IVF, dietary modifications; diarrhea resolved after 12 days
	Female, 59	Adjuvant capecitabine	Severe watery diarrhea (grade 4), palmar-plantar erythrodysesthesia, stomatitis, and severe abdominal pain	CT - diffuse submucosal ileal edema with adjacent fat stranding and pneumatosis intestinalis	IVF, TPN, broad-spectrum antibiotics; diarrhea resolved after 29 days
Mokrim et al., 2014 [10]	Female, 66	Adjuvant capecitabine	Severe watery diarrhea (grade 3), fatigue, emesis, altered mental status	CT - submucosal ileal edema with mural thickening; endoscopy - inflammatory changes in the ileal mucosa; histopathology - inflammatory changes without intraepithelial lymphocytic infiltrates	IVF, broad-spectrum antibiotics; diarrhea resolved after several days
	Female, 67	Adjuvant capecitabine	Severe watery diarrhea (grade 3), fever, fatigue, reduced appetite	CT - parietal thickening of the distal ileal loops	IVF, broad-spectrum antibiotics; diarrhea resolved after several days
Al-Gahmi et al., 2012 [11]	Male, 65	Adjuvant capecitabine with radiotherapy	Watery diarrhea, fever, abdominal pain, vomiting	Endoscopy - isolated ulceration of the terminal ileum; histopathology - inflammatory changes with eosinophilic infiltrate	IVF, conservative treatment; diarrhea resolved after some time
Radwan et al., 2012 [12]	Male, 67	Adjuvant capecitabine	Watery diarrhea (grade 3), reduced appetite, lower abdominal discomfort, and vertigo precipitating falls	CT - distended loops of small bowel, wall thickening, and inflammatory changes	IVF, broad-spectrum antibiotics, TPN, and loperamide; diarrhea resolved after some time
Bouma and Imholz, 2011 [13]	Male, 73	Capecitabine with oxaliplatin and bevacizumab	Watery diarrhea, nausea, vomiting, abdominal pain	CT - ileal mural thickening	IVF, clear liquid diet; diarrhea resolved after several days

The average age of the patients was 64 (range 42-73), and of the 13 patients, nine were female, and four were male. In most cases, capecitabine was administered for either metastatic breast or colon cancer, while in a minority of cases, the indication was primary but locally advanced rectal or colon adenocarcinoma. The clinical picture was dominated by gradually increasing frequency of bowel movements with loosening of stool consistency accompanied by nausea, vomiting, and abdominal pain. In five cases and three cases, the diarrhea was grade 3 and grade 4, respectively. Bloody diarrhea was noted in one patient. In two patients, the degree of dehydration was severe enough to precipitate altered mental status and vertigo with falls. In two patients, concomitant occurrence of terminal ileitis and palmar-plantar erythrodysesthesia was noted, suggesting a potential hypersensitivity reaction. Typical workup began with excluding infectious causes of diarrhea, and in eight patients, prophylaxis with broad-spectrum antibiotics was initiated.

Endoscopic studies may be helpful in further supporting the diagnosis, especially in evaluating potential anastomotic complications in patients who underwent colorectal surgery. Typical findings included ileal ulceration with associated inflammatory changes - in one patient, ulceration was severe enough to precipitate focal hemorrhages with pseudomembrane formation. A cause of refractory CRD is neutropenic enterocolitis, which precludes endoscopic investigations - although evident, potential contraindications must be respected before proceeding with invasive studies in patients taking capecitabine. Reported histopathologic findings include inflammatory changes, eosinophilic infiltrate, mucosal erosion with necrotic debris, without the presence of intraepithelial lymphocytic infiltrates, granulomas, or viral inclusions.

Approach to management presented as a clinical challenge, and in our review, the treatment strategy differed across studies. Three patients were managed with intravenous fluids (IVFs) and bowel rest - dietary modifications (clear liquid diet or low-lactose, low-fat, high-protein diet) appeared to contribute to recovery. Four patients were managed with IVFs and broad-spectrum antibiotics without antidiarrheal therapy. The rest of the patients were managed with IVFs, usually broad-spectrum antibiotics, and some form of step-up antidiarrheal therapy depending on response and other clinical symptoms - loperamide, diphenoxylate-atropine, octreotide, and budesonide. In one patient who previously underwent a right hemicolectomy, cholestyramine was also trialed to address a possible biliary etiology of diarrhea - however, the patient did not improve. Empiric antibiotics were typically discontinued after infectious etiology of the diarrhea was ruled out and instead replaced with antidiarrheal therapy, which few patients responded to adequately. Two patients required total parental nutrition to correct a protein deficiency.

Resolution time of gastrointestinal upset varied from several days up to four weeks, and it is unclear if the severity and the protracted recovery courses are related to the cumulative dose of capecitabine received. Regardless, the backbone of managing these patients included the withdrawal of capecitabine. The safety of resuming capecitabine after recovery is unclear - five patients went on to restart capecitabine (two patients at a reduced dosage), with acceptable tolerability of the regimen. For the rest of the cases, the managing physicians were wary of provoking repeat complications and chose to switch to alternative regimens.

Nicosia et al. described a patient who developed capecitabine-induced terminal ileitis during his 16th fraction of a pelvic irradiation protocol [8]. Within the combinatorial radiochemotherapy treatment regimen, the administered dose of capecitabine was lower than in other cases, leading the authors to speculate that pelvic irradiation may increase the risk of developing ileitis in patients receiving neoadjuvant capecitabine. In a retrospective review of 188 patients who received abdominal or pelvic radiotherapy, Daly et al. found the incidence of chronic ileitis to range from 2.2% to 14.3%, possibly due to radiation enteritis [14]. In order to mitigate this rare complication, meticulous identification and constrained contouring of organs at risk is especially critical to ensure both safety and efficacy of treatment delivery. Al-Gahmi et al. also described ileitis in a patient on combinatorial therapy, but radiation-induced ileitis was ruled unlikely due to low exposure of the ileum to the radiation field [11].

The mechanism behind capecitabine-induced diarrhea, and potentially ileitis, is complex and multifaceted. As mentioned previously, early studies identified capecitabine causing mitotic arrest of crypt cells in the G2 phase, impairing their subsequent migration and differentiation into mature enterocytes [5]. The colon's diminished absorptive capacity works in tandem with goblet cell hyperplasia and subsequent excessive mucin secretion from the small bowel to cause secretory diarrhea. However, the mechanism may be considerably more complex, as fluoropyrimidines may increase gene expression of inflammatory cytokines and cause decreased expression of colonic aquaporin channels through neutrophilic inflammation [15]. Mitotic arrest in smooth muscle cells, leading to apoptosis and reduction in contractility through actin cytoskeleton reorganization, has also been documented [16]. Finally, fluoropyrimidines are directly toxic to the endothelium through increased generation of reactive oxygen species - this toxicity can lead to thrombosis or vasospasm via release of sequestered vasoactive substances [17]. It is conceivable that all of these pathologic processes contribute to the clinical phenotype.

Capecitabine is metabolized to the active metabolite 5-FU through several enzymatic reactions, which preferentially occur in solid tumors and the liver. Genetic polymorphism of drug-metabolizing enzymes through the variable expression of single nucleotide (SNPs) is well described and may explain why only select patients suffer from adverse drug reactions, such as terminal ileitis. The majority of 5-FU is catabolized by DPD, which is encoded by the highly polymorphic DPYD gene [18]. Deficiency of DPD is reported in approximately 5-9% of patients, and administering fluoropyrimidines in the context of depressed enzyme activity can be fatal [4,19].

In our review, DPD genotype testing was done in three patients, and a mutation was found in two of them. In both cases, there was only a partial reduction in DPD activity. Curiously, the patient described by Mokrim et al. only developed symptoms of ileitis after completing an entire cycle of capecitabine despite having diminished DPD activity [10]. Recommendations are in place advocating for routing screening for four most common DPD variants before initiating treatment with capecitabine, especially in regions with accentuated prevalence [19]. A recent genome-wide association study identified three novel SNPs, which can be used as germline genetic predictors of capecitabine-associated diarrhea and, potentially, ileitis [20]. The toxicity capture approach was used in patients being treated with capecitabine for breast cancer, with further research being required to identify if cancer type influences SNP effects on enzyme activity and adverse drug event incidence. The application of genetic screens in identifying patients who are likely to develop capecitabine toxicity is promising and may aid in reducing morbidity.

## Conclusions

Capecitabine-induced terminal ileitis is rare but may be life-threatening - treating physicians should be aware of this potential complication and intervene early when persistent, high-grade diarrhea is reported. CT scans showing edematous mural thickening and distention of the terminal ileum should raise suspicion for ileitis. The cornerstone of conservative management includes the withdrawal of capecitabine, antidiarrheal therapy, and broad-spectrum antibiotics, and dietary modifications are acceptable treatment modalities. Pelvic irradiation may represent a risk factor, which increases the likelihood of developing terminal ileitis with capecitabine. Restarting capecitabine may be attempted if the patient harbors normal DPD function, although further research is required to validate safety outcomes. 
